# Unveiling the multi-step solubilization mechanism of sub-micron size vesicles by detergents

**DOI:** 10.1038/s41598-019-49210-0

**Published:** 2019-09-09

**Authors:** Paul A. Dalgarno, José Juan-Colás, Gordon J. Hedley, Lucas Piñeiro, Mercedes Novo, Cibran Perez-Gonzalez, Ifor D. W. Samuel, Mark C. Leake, Steven Johnson, Wajih Al-Soufi, J. Carlos Penedo, Steven D. Quinn

**Affiliations:** 1 SUPA School of Physics and Astronomy, University of St. Andrews, North Haugh, Fife, KY16 9SS UK; 2 Institute of Biological Physics and Bioengineering, School of Engineering and Physical Sciences, Heriot-Watt University, Edinburgh, EH14 4AS UK; 3 Department of Electronic Engineering, University of York, Heslington, York, YO10 5DD UK; 4 Department of Physical Chemistry, Faculty of Science, University of Santiago de Compostela, Lugo, E-27002 Spain; 5 Department of Physics, University of York, Heslington, York, England YO10 5DD UK; 6 Department of Biology, University of York, Heslington, York, YO10 5DD UK; 7 Biomedical Sciences Research Complex, University of St. Andrews, North Haugh, St. Andrews, Fife KY16 9ST UK; 8 Present Address: School of Chemistry, University of Glasgow, Glasgow, Scotland G12 8QQ United Kingdom

**Keywords:** Fluorescence imaging, Single-molecule biophysics

## Abstract

The solubilization of membranes by detergents is critical for many technological applications and has become widely used in biochemistry research to induce cell rupture, extract cell constituents, and to purify, reconstitute and crystallize membrane proteins. The thermodynamic details of solubilization have been extensively investigated, but the kinetic aspects remain poorly understood. Here we used a combination of single-vesicle Förster resonance energy transfer (svFRET), fluorescence correlation spectroscopy and quartz-crystal microbalance with dissipation monitoring to access the real-time kinetics and elementary solubilization steps of sub-micron sized vesicles, which are inaccessible by conventional diffraction-limited optical methods. Real-time injection of a non-ionic detergent, Triton X, induced biphasic solubilization kinetics of surface-immobilized vesicles labelled with the Dil/DiD FRET pair. The nanoscale sensitivity accessible by svFRET allowed us to unambiguously assign each kinetic step to distortions of the vesicle structure comprising an initial fast vesicle-swelling event followed by slow lipid loss and micellization. We expect the svFRET platform to be applicable beyond the sub-micron sizes studied here and become a unique tool to unravel the complex kinetics of detergent-lipid interactions.

## Introduction

Detergent-induced membrane solubilization is critical for applications including membrane-protein purification^1,2^, and targeted drug delivery, where vesicle rupture enables release of encapsulated therapeutics^3^. Despite decades of widespread use, the complexity of membrane solubilization, coupled with limitations in current technology, have made characterizing its mechanism extremely challenging^4^.

Initial biochemical experiments indicated that the rate of membrane solubilization depends on the lipid phase, and the type and concentration of detergent^5^. The non-ionic detergent Triton X 100 (TX-100), for example, solubilizes phosphocholine (PC) rich membranes relatively slowly below the gel-to-liquid transition temperature but speeds up rapidly in the fluid phase^6,7^. In the gel state, however, the concentration of detergent required to achieve complete solubilization is strongly dependent on the lipid chain length^8^. For most biochemical applications, TX-100 is the solubilizer of choice, and is used as a reference for measuring the activity of other surfactants^9,10^. Turbidity measurements also reported the TX-100: lipid ratios required to solubilize lipid vesicles as a function of phase^11–13^ and lipid^14^, and isothermal titration calorimetry has probed the initial TX-100-membrane interaction^15^. These experiments suggest an interplay between surfactant monomers and lipids at the detergent’s critical micellar concentration (CMC) in which lipid re-arrangement leads to heat transfer and mixed-micelle formation within the intact membrane^16^.

Importantly, the solubilization activity of TX-100 is inhibited by membrane cholesterol, though the precise mechanism is still unclear. Cholesterol may initiate liquid ordered, detergent-resistant regions across the membrane^12,17^, but whether these are microdomains^18^, detergent-resistant rafts^19^ or a combination of both^20^ requires confirmation. Alternatively, TX-100 may promote liquid-ordered phases via interaction with order-preferring cholesterol-rich regions rather than initiating lipid reorganization^21^.

Despite its complexity, the most widely adopted model to describe membrane solubilization is the three state mechanism^22^. In State 1, detergent monomers partition the bilayer until a saturation value (R_sat_) is reached^23,24^. This results in an increase in mass and an increase in turbidity due to vesicle swelling, and potentially, fusion between vesicles. In State 2, the membrane starts to disintegrate, and this phase involves the formation of mixed detergent-lipid micelles coexisting with the bilayer. State 3 corresponds to breakdown of the membrane into mixed-micelles in solution. There are, however, many unanswered questions, particularly regarding the timescales of these processes mostly due to a lack of methods that can unambiguously dissect each stage of the solubilization process. While Cryo-TEM, NMR and conventional dynamic light scattering all provide snapshots of the membrane conformation^25–27^, they cannot provide dynamic insight. Conversely, ITC probes the thermodynamics and turbidity measurements reveal solubilization conditions, but neither reveal structure^28,29^. Molecular dynamics simulations have attempted to bridge this gap^17,30,31^ and coarse-grained simulations reveal a sequence of events in broad agreement with the three-state mechanism^10^. Additionally, phase contrast and fluorescence microscopy have also been used to study the solubilization mechanism of giant (10–20 μm) unilamellar vesicles (GUVs) and the influence of cholesterol^11^. These studies demonstrated that alterations in vesicle shape following the injection of non-ionic TX-100 or anionic sodium dodecyl sulfate (SDS) are regulated by the very different flip-flop rate of both detergents. For TX-100, an almost instantaneous flip-flop rate (<0.5 s) ensures equilibration of detergent molecules across both leaflets^5,13,32^ that results in swelling and an increase in surface area of the GUVs. In these studies, the formation of pores in the PC bilayer leads to complete solubilization into micelle-like structures for TX-100 concentrations of ~0.18 mM, well below the detergent CMC (~0.28 mM). In contrast, SDS exhibits a flip-flop rate in the range of minutes to hours at room temperature, and a concentration of SDS (~30 mM) much higher than its CMC (~8 mM) was needed to induce solubilization and no increase in surface area was observed. These studies confirmed a stepwise solubilization mechanism of GUVs by both detergents and demonstrated that the structures adopted by the lipid-detergent complex are strongly influenced by the ability of the detergent to rapidly equilibrate between both leaflets. Unfortunately, conventional techniques such as phase contrast and fluorescence microscopy used in these studies can only resolve changes in shape for large objects with diameters usually above ~5–10 μm, which constitutes only one end of the curvature space of lipid membranes. Furthermore, traditional optical microscopy quantifies macroscopic changes in size and packing density but provides little structural information at the molecular level. Therefore, there is a need to develop complementary methods that can monitor the solubilization process of individual vesicles smaller than the diffraction limit (~250 nm) that constitute the other end of the membrane curvature space, and which are commonly used in biotechnological applications but cannot be studied by conventional microscopy.

In this work we demonstrate the combination of single-molecule fluorescence and Förster resonance energy transfer (FRET) to monitor, in real-time, the detergent-induced solubilization of large unilamellar vesicles (LUVs) with sizes smaller than the diffraction limit. FRET is sensitive to 1–10 nm distances between two small organic dyes termed donor (D) and acceptor (A)^33^ and in svFRET, lipophilic fluorophores are incorporated directly within the membrane to act as reporters of molecular interactions. Although svFRET has been applied to investigate the kinetics of membrane fusion^34,35^ and pore formation^36^, its application for characterizing solubilization kinetics has not been reported. Importantly, by immobilizing individual vesicles on the surface of a microscope slide via biotin-streptavidin interactions, structural changes in the lipid vesicle can be monitored without interference from vesicle fusion. We demonstrate that by monitoring the time-dependent variations in FRET efficiency (E_FRET_) and total emission intensity (I_D_ + I_A_) following the addition of detergent provides a means to differentiate, for the first time, each structural step along the solubilization process and unambiguously extract the rates of swelling and lysis events as a function of detergent concentration and cholesterol content.

Solubilization profiles of LUVs of sizes between 100 nm and 200 nm at concentrations of TX-100 near the critical micellar concentration (CMC_Tx-100_ ~ 0.28 mM) were characterized by a rapid increase (~5 s) in vesicle surface area, reported by a pronounced decrease in FRET efficiency with no change in total intensity. This swelling step is followed by a slow lysis phase (~40 s) involving loss in lipid content that results in a remarkable decrease in total intensity without significant variation in FRET efficiency. By measuring the diffusion coefficient of labelled LUVs using fluorescence correlation spectroscopy (FCS), we estimated a 34% increase in vesicle size induced by TX-100 and confirmed that the observed decrease in E_FRET_ on immobilized LUVs reflects vesicle swelling. Quartz-crystal microbalance with dissipation (QCM-D) revealed a 5% mass gain during the first few seconds after TX-injection followed by a 63% mass loss at the final stages of solubilization with timescales comparable to those observed by svFRET, thus confirming the assignation of each event in the svFRET trajectories. When the same experiments were carried out in the presence of 20% cholesterol, we observed a remarkable decrease in the rate of swelling with much smaller impact on the lysis rate, suggesting that the previously reported resistance to solubilization conferred by cholesterol to PC vesicles might arise from cholesterol inhibiting the initial step of detergent insertion in the lipid bilayer. In summary, we demonstrate the use of a multi-disciplinary approach combining the novel application of svFRET to dissect the elementary stages of the solubilization process and extract kinetic information with FCS and QCM-D to quantify changes in size and mass of sub-micron size vesicles with high membrane curvature. Given that membrane curvature is emerging as an important mechanism regulating the recruitment of numerous proteins and peptides^37^, the svFRET technique should become an exceptional tool that complements current optical microscopy and phase contrast methods when targeting the entire curvature space of lipid bilayers.

## Results and Discussion

**Figure 1:**
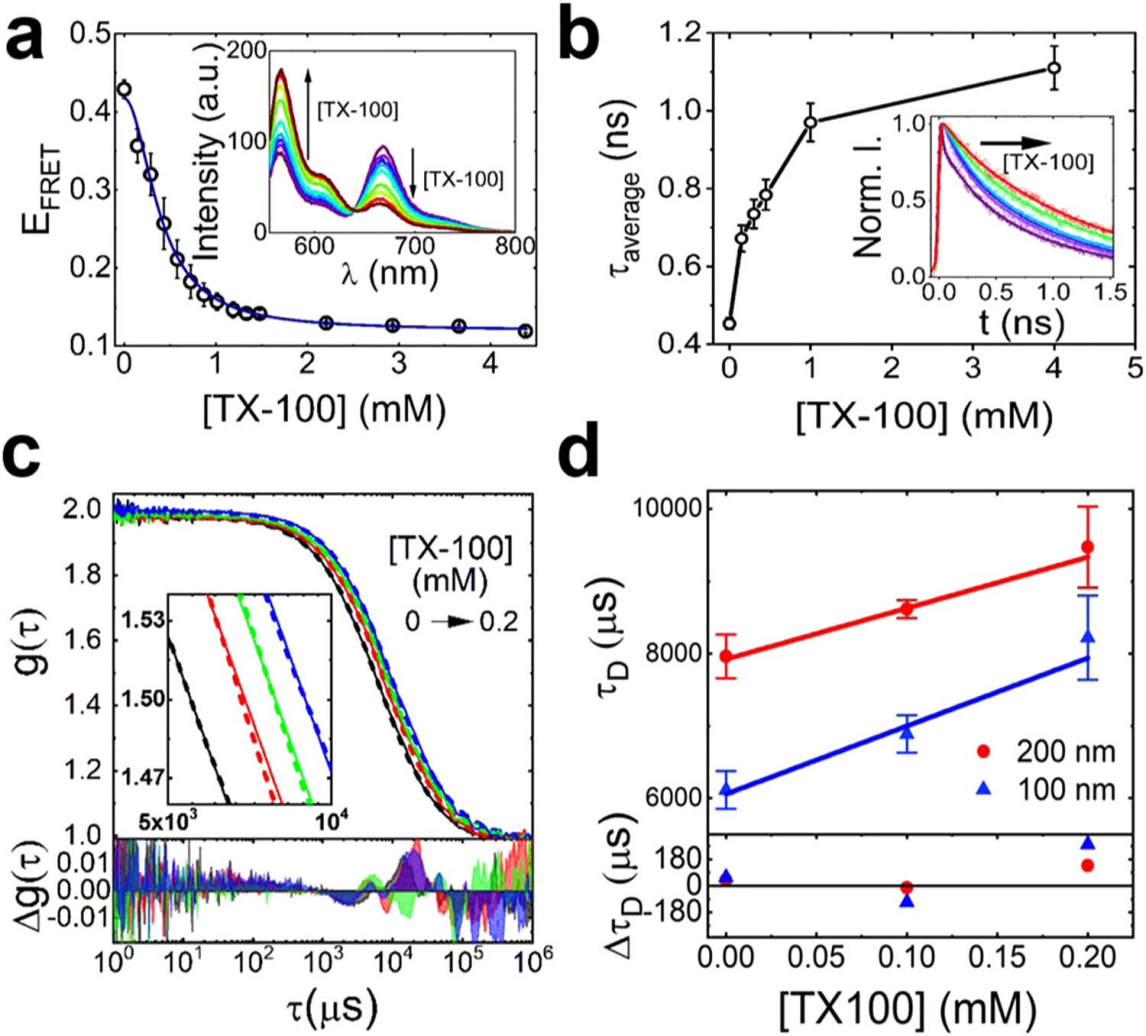
TX-100 vesicle interactions reported using ensemble FRET and FCS. (**a**) FRET efficiency of Dil/DiD labeled vesicles versus TX-100. The solid line represents a Hill model fit. Inset: corresponding variation in fluorescence spectra. (**b**) Average lifetime of Dil versus TX-100. Inset: corresponding time-resolved fluorescence decays. Solid lines represent bi-exponential fits. (**c**) Top: FCS cross-correlation curves (solid lines), fits (dashed lines) (inset: zoomed in) associated with 100 nm-(black) and 200 nm-sized (red) vesicles. Also shown are FCS curves for 200 nm-sized vesicles in the presence of 0.1 mM (green) and 0.2 mM (blue) TX-100. Bottom: residuals of the fits. (**d**) Top: Diffusion times of NBD-PC labeled vesicles as a function of TX-100. A PC: PS lipid ratio of 65: 35 was used under all conditions. Solid lines represent linear fits. Bottom: corresponding residuals.

PC- and phosphoserine (PS)-rich model-membrane vesicles incorporating 20% cholesterol were prepared as detailed in the Methods and are schematically shown in Fig. S1. The amounts of donor (Dil) and acceptor (DiD) per vesicle were optimized (1:1, 0.1% of each dye) such that the average FRET efficiency (E_FRET_) per vesicle was initially close to 0.5, enabling nanometer length scale changes to be quantified by an observable change in E_FRET_ in either direction. The production of homogeneously distributed unilamellar vesicles (d ~ 200 nm) was confirmed by dynamic light scattering (Fig. S2). Steady-state fluorescence measurements were carried out as an initial step to characterize the interaction between TX-100 and labelled vesicles. As the concentration of TX-100 was progressively increased, we observed a decrease in E_FRET_ (Fig. 1a), from a value of 0.43 ± 0.05 in the absence of TX-100, to 0.13 ± 0.02 in the presence of 4.4 mM with a half-maximal concentration constant of 0.39 ± 0.07 mM. This data suggests that the addition of TX-100 induces changes in vesicle structure, or composition, that results in a high distance separation between the dyes. The decrease in E_FRET_ was further confirmed by time-correlated single photon counting, where the amplitude weighted average lifetime of Dil progressively increased as a function of TX-100 (Fig. 1b and Table S1).

Having established FRET as a sensor of fluorophore separation in the ensemble, fluorescence correlation spectroscopy (FCS) was used to probe the diffusion of single vesicles. The high sensitivity of FCS to the size of diffusing vesicles makes it an attractive technique for accessing their diameter under solubilizing conditions. Interestingly, the use of FCS techniques has mainly focused on the formation of micelle-like structures, the understanding of vesicle fusion and protein-lipid interactions, but their application in the context of lipid-detergent interactions remains under-explored^38–40^.

The translational diffusion times of vesicles prepared with 0.2% (1-palmitoyl-2-{6-[(7-nitro-2-1, 3-benzoxadiazol-4-yl) amino] hexanoyl}-*sn*-glycero-3-phosphocholine) (NBD-PC) (Fig. S3), a fluorescent analog of PC, were recorded. NBD-PC replaced Dil and DiD for compatibility with the apparatus. Normalized cross-correlation functions obtained from freely-diffusing vesicles progressively shifted towards longer diffusion times as a function of TX-100 (Fig. 1c). In the absence of detergent, vesicles of diameters ~100 nm and ~200 nm displayed diffusion times, τ_D_, across the confocal volume of 6.1 ± 0.03 ms and 8.0 ± 0.3 ms, respectively (Fig. 1d). τ_D_ associated with the diffusion of the smaller vesicles increased by ~13% in 0.1 mM TX-100 and by ~34% in 0.2 mM TX-100. The larger 200 nm-diameter vesicles also displayed a similar trend, representing an ~8% and ~18% increase in hydrodynamic diameter at 0.1 mM and 0.2 mM TX-100, respectively. These data point toward an increase in mean vesicle diameter when incubated with TX-100, and was attributed to vesicle expansion, fusion or a combination of both in solution. It is interesting to note that the relative increase in τ_D_ and the resulting increase in size (Table S2) are slightly higher for smaller LUVs and that this trend is maintained at the two concentrations of TX-100 investigated. This results in a 25% higher slope for the 100 nm LUVs (9.40 ± 1.4) × 10^3^ µs mM^−1^ compared to (7.0 ± 1.4) × 10^3^ µs mM^−1^ for the 200 nm LUVs (Fig. 1d). We interpreted this as evidence that the degree of swelling depends on vesicle size; an observation that emphasizes the importance of membrane curvature modulating the initial stages of TX-100 insertion in the lipid bilayer. As shown in recent molecular dynamics studies, the formation of lipid packing defects is intimately linked to membrane curvature in addition to lipid composition^41^ and we hypothesize that the higher slope observed for the smaller vesicles might reflect the formation of stress-induced defects that facilitate the insertion of detergents. This is an interesting finding to consider for further studies because many natural and synthetic compounds must partition in the lipid bilayer to reach their targets.

**Figure 2:**
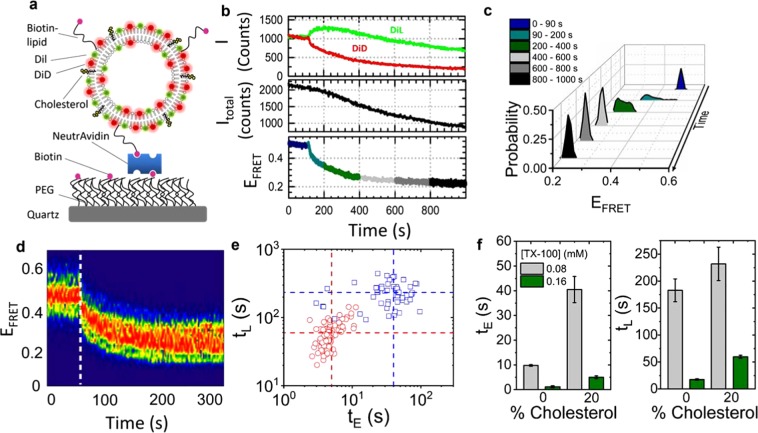
Real-time visualization of solubilization kinetics by svFRET. (**a**) Schematic of the immobilization scheme. The schematic is for illustration purposes only and is not to scale. (**b**) Representative variation in the fluorescence emission of Dil and DiD (top panel), the sum of their intensities (middle panel) and the corresponding variation in FRET efficiency obtained before (<90 s) and after (>90 s) injection of 0.16 mM TX-100. (**c**) Relative FRET state occupancies observed over 1000 s. (**d**) FRET contour plot showing the variation in E_FRET_ before and after TX-100 injection (dashed white line) (N = 105). (**e**) Corresponding scatter plot of expansion half-live, t_E_ versus that of lysis, t_L_ obtained after injection of 0.08 mM (blue) and 0.16 mM (red) TX-100. Dashed lines represent the center of each distribution. (**f**) Comparative bar plots summarizing the variation in t_E_ and t_L_ as a function of TX-100 and percentage of cholesterol incorporated within the vesicle bilayer. Error bars indicate the standard error of the mean.

In the next step, to rule out the possibility of fusion and investigate in more detail each step of the solubilization process, Dil/DiD labelled vesicles containing a low percentage of biotinylated lipids and 20% cholesterol were immobilized onto a NeutrAvidin-coated surface and imaged via total internal reflection fluorescence microscopy. As illustrated in Fig. 2a, biotinylated vesicles were anchored to NeutrAvadin tethered to the surface via biotinylated polyethylene glycol (PEG). In the absence of TX-100 the vesicles were stable with no variation in the svFRET efficiency observed (Fig. 2b). Perturbation of single vesicles by TX-100 was then reported as observable changes in the svFRET efficiency in real-time with 50 ms time integration (Fig. 2b,c). To suppress photobleaching and optimize conditions for svFRET, the fluorescence response of single vesicles labelled with DiD were investigated as a function of excitation intensity and percentage of dye-loading content. As demonstrated in Supplementary Text I, Fig. S4 and Table S3, excitation intensities <0.04 mW/cm^2^ with 0.25% dye were necessary for long-term (180 s) stability of the incorporated dyes. In the absence of TX-100, the FRET efficiency from single vesicles remained largely invariant with a value of E_FRET_ ~ 0.5. Injection of 0.16 mM TX-100 then induced variations in E_FRET_ and total intensity on remarkably different time scales. Immediately after injection of TX-100, the FRET efficiency decreased from a value of ~0.46 to ~0.22 in a 250 sec time window (Figs 2d and S5). Most of this change happened in the first 100 seconds after injection, pointing towards a ~20% increase in the separation distance between FRET pairs. Assuming spherical vesicles, this distance scales directly with the vesicle radius and thus agrees well with the FCS data. Importantly, within this time window, the total intensity decreased only by ~7%. At time scales longer than 250 seconds, the FRET efficiency did not change further, whereas the total intensity decreased progressively to ~50% of its initial value. The different timescales and responses of both signals suggest that they represent different distortions of the vesicle structure. The rapid decrease in FRET efficiency without significant variation in total intensity indicates a structural change involving no loss of lipid content and we assigned it as arising from vesicle swelling induced by TX-100 molecules inserting into the lipid bilayer and increasing the average inter-dye distance. The time window where the FRET efficiency does not change but the total intensity is strongly decreased suggests a structural distortion involving the diffusion of lipids into solution and it was assigned to a lysis step resulting in the formation of micelles. The FRET efficiency plateau value observed in this time window (E ~ 0.22) represents an inter-dye distance ~6.5 nm within these micelles. These observations point towards a fast vesicle expansion event with half-life t_E_, followed by a slower lysis event (t_L_). The expansion step was observed to occur on average 12 times faster than lysis, and at 0.08 mM TX-100, the half-lives associated with each event increased by ~75% (Fig. 2e). The ability to unambiguously discriminate between expansion and lysis events and extract individual kinetic rates for each stage during LUV solubilization is a completely new finding only afforded by the development of svFRET for surface-immobilized vesicles.

The observation of a stepwise solubilization mechanism in LUVs qualitatively agrees with optical microscopy and phase contrast measurements reported in GUVs composed of PC and sizes of ~20–30 µm^11,12^. In these studies, it was found that the addition of sub-CMC concentrations of TX-100, in the range of those used in our work, resulted in an increase in vesicle diameter a few seconds after injection. Increasing further the concentration of TX-100 to near CMC values resulted in dynamic perforation of the bilayer leading to complete solubilization in a timescale of ~20–30 seconds. Importantly, when SDS was used as the detergent, no increase in surface area was detected and the solubilization process was slow and only efficient at concentrations well above the CMC (8 mM). It was suggested that the different rates of detergent equilibration between the two leaflets of the bilayer may govern the solubilization process and dictate the morphological changes taking place in the vesicle during the initial stages. A direct quantitative comparison between our findings on LUVs and the previous work on GUVs is not straightforward for several reasons. First, as discussed in those studies, optical microscopy and phase contrast have access only to the cross-section of the focal plane of the vesicle and thus quantification of surface area in vesicles that might adopt non-trivial shapes becomes unfeasible. Secondly, given the size of the GUVs and the injection of detergent using a micropipette, the concentration of detergent around the vesicle is not homogeneous, the vesicle deforms more rapidly on the side facing the micropipette, and this might influence the overall kinetics of the process. In contrast, for the immobilized LUVs used in our study, filling the entire microchannel takes place in ~1 second and the detergent flow wave front should achieve a steady-state concentration much faster than any of the processes investigated. Nevertheless, it is clear that LUVs and GUVs shared some common solubilization features by TX-100. This includes similar concentration requirements and the presence of an initial and rapid expansion step followed by a slower step where lipids are released to the solution to form micelle-like structures. Thus, our svFRET studies on highly curved sub-micron size vesicles are complementary to optical microscopy on giant vesicles and both suggest a common general mechanism of solubilization.

Since cholesterol alters the membrane structure^42^, we next employed svFRET to assess the influence of cholesterol on the stability and kinetic mechanism of vesicle solubilization. 200 nm diameter vesicles were prepared in the absence of cholesterol and were induced to solubilize by 0.08 mM TX-100. Here, the expansion and lysis half-lives reduced by ~76% and ~21% respectively, compared with vesicles loaded with 20% cholesterol. When the TX-100 concentration was doubled, t_E_ and t_L_ reduced further to 1.2 ± 0.4 and 17.6 ± 1.6 seconds, respectively, as shown via a comparative bar plot summarizing the relative variations in t_E_ and t_L_ as a function of cholesterol and TX-100 concentration (Figs 2f and S6). To the best of our knowledge, these values constitute the first direct measurement on the effect of cholesterol on each individual step of the solubilization process. The relatively small decrease observed for the kinetic rate of the lysis step in the presence of 20% cholesterol compared to that with no cholesterol added agrees with previous observations on binary PC/Cholesterol mixtures where no significant effect on the solubilization rate was found at any temperature using turbidity measurements^43^. In contrast, the remarkable decrease in the timescale for vesicle swelling in the presence of cholesterol was not detected in previous turbidity measurements but agrees with recent phase contrast and light scattering observations on a similar lipid bilayer composition^11,44^. In these studies, a significant decrease in the surface area variation during the swelling step was reported from a value of 48% with no cholesterol to ~32% in the presence of 30% cholesterol. Because the swelling event reflects mostly the initial interaction between TX-100 and the lipid membrane, the slower rate of swelling in the presence of cholesterol can be interpreted as a decrease in the association rate of TX-100 to the lipid bilayer. A decrease in the association rate of TX-100 aligns well with the reported decrease in binding constant induced by cholesterol in PC vesicles^11^ and potentially reinforces the curvature-mismatch hypothesis^27^ whereby the incorporation and packing of cholesterol is incompatible with the association of TX-100 molecules due to their opposite curvature.

**Figure 3:**
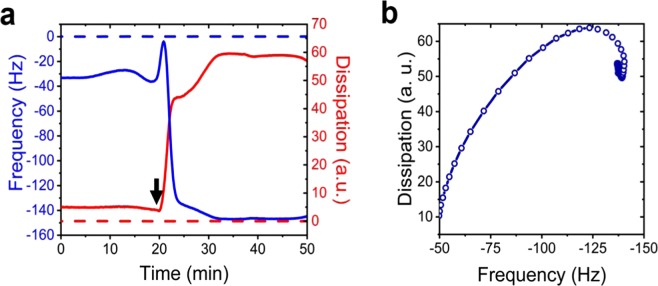
TX-100 induced vesicle solubilization monitored by QCM-D. (**a**) Variation in frequency (blue) and dissipation (red) of the 7^th^ overtone associated with surface immobilized vesicles in the presence of TX-100. The dashed lines represent data collected from a control sensor pre-treated with PEG and NeutrAvidin, but lacks vesicles. The arrow indicates the time-point of the solubilization. (**b**) Frequency versus dissipation observed during the interaction between surface immobilized vesicles and TX-100.

**Figure 4:**
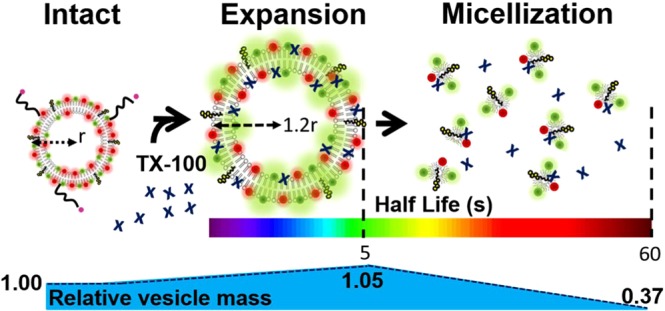
Mechanism of TX-100 induced vesicle solubilization. Detergent molecules approach lipid vesicles inducing a fast conformational expansion prior to lysis and the release of mixed-micelles into solution.

The svFRET and FCS experiments discussed so far have provided new structural and kinetic information regarding the solubilization mechanism of LUVs by TX-100. However, given that the main consequence of swelling and lysis events is the transfer of mass from the vesicle to solution and *vice* v*ersa*, a complete description of the mechanism will benefit from the development of methods that can evaluate such mass gain or loss at each stage of the process. To quantify the transfer of mass during solubilization, we employed a label-free quartz-crystal microbalance with dissipation (QCM-D) monitoring approach (Supplementary Text II). QCM-D has recently emerged as a very useful method to monitor vesicle fusion^45^, vesicle adsorption to surfaces^46^ and protein-vesicle interactions^47^ but its application in the context of detergent-induced solubilization has not been reported. Here, PC/PS vesicles containing 20% cholesterol were immobilized onto a quartz sensor surface and interactions with 0.16 mM TX-100 were followed by changes in oscillation frequency and dissipation, reflecting mass and viscoelasticity on the sensor surface, respectively (Fig. 3a). Interactions between immobilized vesicles and TX-100 were observed via changes in both frequency and dissipation traces immediately after TX-100 injection corresponding to a ~5% mass gain at the sensor which we attributed to TX-100 incorporation into vesicles. This was followed by an interaction that leads to a conformational change in intact vesicles, with no mass loss, over the first 35 s. As the local TX-100 concentration then increased, the deposited mass accumulated on the surface leading to a decrease in resonance frequency. A substantial mass loss of ~63% was then observed via an increase in resonance frequency, indicating material immobilized to the surface was released into solution (Fig. 3b). These processes occurred on similar timescales to those obtained under the same conditions using svFRET and control experiments performed simultaneously indicated no interaction with the PEG-coated sensor surface and TX-100 (Fig. 3a). These findings support a mechanism through which TX-100 accumulates on the curved membrane surface, preceding a rapid expansion of the vesicle structure that, in turn, precedes a slower lysis event (Fig. 4).

In summary, we have directly monitored the solubilization of sub-micron size lipid vesicles that cannot be resolved using conventional optical techniques in response to TX-100 using svFRET, FCS and QCM-D. We show that the combination of svFRET and surface-immobilization of LUVs is a unique method to discriminate between the swelling and lysis steps and unambiguously quantify the kinetic rates of each process without interference from vesicle fusion. Our data demonstrate that detergent-induced swelling is a relatively fast process that is strongly slowed down by the presence of cholesterol whereas the subsequent lysis step is only marginally affected. The increase in vesicle size during swelling was calculated using FCS and suggested that increasing the membrane curvature facilitates the insertion of detergent molecules. Our assignation and characterization of the timescales for the swelling and lysis steps by svFRET and FCS was further supported by quantifying the mass gain (swelling) or loss (lysis) using QCM-D measurements. Understanding the structural stability and dynamics of small vesicles with high curvature is crucial not only because they are commonly employed in biotechnological applications but also because important traffic pathways between the endoplasmic reticulum rely on the formation of LUVs and a large fraction of it consists of tubules of ~30 nm radius^48^. In the future, the experimental approach presented here may be useful in several directions: to quantify the effect of membrane curvature on each step of the solubilization process, to determine the role of lipid composition on the solubilization of LUVs and to quantify the interaction with small molecules that need to target and cross the lipid membrane.

## Methods

### Materials

1-palmitoyl-2-oleoyl-sn-glycero-3-phospho-L-serine (sodium salt) (PS), 1-palmitoyl-2-oleoyl-glycero-3-phosphocholine (PC) and 1,2-dioleoyl-sn-glycero-3-phosphoethanolamine-N-(cap biotinyl) (sodium salt) (biotinylated lipid) phospholipids were purchased from Avanti Polar Lipids Inc. 1,1′-Dioctadecyl-3,3,3′,3′-Tetramethylindocarbocyanine Perchlorate (Dil) and 1,1′-Dioctadecyl-3,3,3′,3′-Tetramethylindodicarbocyanine, 4-Chlorobenzenesulfonate Salt) (DiD) membrane stains were purchased from ThermoFisher Scientific.1-palmitoyl-2-{6-[(7-nitro-2-1,3-benzoxadiazol-4-yl)amino]hexanoyl}-sn glycerophosphocholine (NBD-PC) was purchased from Sigma Aldrich. All phospholipid samples were used without additional purification and stored in chloroform at −20 °C prior to use. Dil, DiD and cholesterol stocks were stored at 4 °C in chloroform prior to use. Triton X-100 was purchased from Sigma Aldrich and freshly suspended in 50 mM Tris (pH 8) prior to use.

### Preparation of large unilamellar vesicles

Mixtures of lipids and lipophilic dyes were homogeneously dispersed in chloroform, dried by nitrogen flow and stored under continuous vacuum pumping at room temperature for 5 hours. Phospholipid mixtures were subsequently re-suspended in buffer solution (50 mM Tris, pH 8) and mixed well by vortex. Large unilamellar vesicles were prepared by the extrusion method at room temperature, in which they were passed through a polycarbonate membrane filter of defined pore size. A molar ratio of 65: 35 PC: PS was used throughout. Vesicles were labelled with dyes (0–0.5%), cholesterol (0–20%) and biotin (1%) at the molar percentages specified in the text. The mean size of the prepared vesicles in solution was evaluated by dynamic light scattering using a Zetasizer μV molecular size detector (Malvern Instruments Ltd., UK).

### Steady-state fluorescence spectroscopy

Fluorescence emission spectra were acquired using a Varian Eclipse fluorescence spectrophotometer. Spectra from Dil and DiD were recorded using an excitation wavelength of 532 nm at magic angle. FRET efficiencies were approximated by the apparent FRET efficiency, E_FRET_ = (I_665_/[I_665_ + I_565_]), where I_665_ and I_565_ represent the fluorescence intensities of the acceptor at 665 nm, and donor at 565 nm, respectively. The FRET efficiency data shown in Fig. 1a was fitted to a Hill model of the form, ${E}_{FRET}=A+B\frac{{[TX-100]}^{n}}{{k}^{n}+{[TX-100]}^{n}},$ where A and B are the measured FRET efficiencies at the start and end of the titration, k is the half-maximal concentration constant and n is the Hill coefficient. The parameters of the fit shown in Fig. 1a are A = 0.43 ± 0.04, B = 0.12 ± 0.01, k = 0.39 ± 0.07 and n = 2.0 ± 0.3 (χ^2^ = 0.99). Error bars represent the standard error of the mean from 3 individual experimental runs.

### Time-resolved fluorescence spectroscopy

Fluorescence lifetime measurements were performed with a Hamamatsu C6860 Synchroscan streak camera. The 80 MHz, 100 fs (full width half maximum) 800 nm output of a Ti: Sapphire oscillator was frequency doubled with a beta barium borate crystal, giving 400 nm excitation pulses. The 400 nm light, with an average power of less than 1 mW, was subsequently focused through the optical path length (1 cm) of the solution cuvette. Fluorescence from the sample was then collected and collimated with a lens before being focused onto the entrance slit of a Chromex 250 is imaging spectrograph. Excitation light was removed with a yellow schott glass filter that cuts all light below 420 nm. A spectral window of 585–607 nm corresponding to Dil fluorescence emission was selected with the streak camera. Time-resolved fluorescence dynamics were then recorded enabling time constants of down to approximately 10 ps to be resolved with instrument response deconvolution.

### Fluorescence correlation spectroscopy

Samples were deposited on glass-bottomed well plates (Whatman) and excited by the linearly polarized light of a 488 nm continuous wave laser (Becker & Hickl) which was spectrally cleaned (Semrock, US, FF01-482/18), redirected by a dichroic mirror (Semrock, US, DI01-R488) and focused into the sample by a 60x water immersion microscope objective (Olympus, UPLSAPO60xW/1.2) mounted in an inverted microscope (Olympus, IX-71). The fluorescence was focused onto a ϕ = 50 μm pinhole (Thorlabs) before being split by a 50:50 nonpolarizing beamsplitter cube (Thorlabs). Each beam was then focused onto an avalanche photodiode (MPD50CTC APD, ${\varnothing }$ = 50 μm, Micro Photon Devices). An emission filter (Semrock, 525/45) placed in front of the beamsplitter was used to discriminate fluorescence from scattered light. The detector signals were processed and stored by two time-correlated single photon counting (TCSPC) modules (Becker & Hickl, SPC 132). Typically 20 million photons were collected for each correlation curve with count rates between 5 and 20 kHz. All measurements were made at a stabilized temperature of 25.0 ± 0.5 °C. The excitation power as measured in the focus of the microscope objective by a power meter (Thorlabs) was 0.02 mW corresponding to a mean irradiance of 7.15 kW/cm^2^ assuming a Gaussian intensity distribution along the optical axis. The focal area and the detection volume were calibrated with Rhodamine 123 in aqueous solutions at low irradiance using an estimated diffusion coefficient of 4.6 ± 0.4 × 10^−10^ m^2^ s^−1^, yielding a radial 1/e^2^ radius of ω_xy_ = 0.27 μm and volume of focus of V = 0.53 μm^3^. Correlation functions were calculated according to $G=\frac{\langle I(t)+I(t+\tau )\rangle }{\langle I{(t)}^{2}\rangle }$ where I(t) is the intensity at time t and fitted according to $G={b}_{0}+\frac{1}{N}{(1+\frac{t}{{\tau }_{D}})}^{-1}\sqrt{{(1+\frac{t}{{\Omega }^{2}{\tau }_{D}})}^{-1}}(1+{A}_{T}{e}^{-t/{\tau }_{T}})$ where N is the number of molecules, t is the correlation time, A_T_ is the amplitude of the triplet and τ_T_ is the triplet time. Ω defines the ratio between the axial and radial 1/e^2^ radii, ω_z_ and ω_xy_, respectively: $\Omega =\frac{{\omega }_{z}}{{\omega }_{xy}}$. Translational diffusion coefficients, D, were determined by $D=\,\frac{{\omega }_{xy}^{2}}{4{\tau }_{D}}$ where τ_D_ is the diffusion time. All diffusion coefficients were corrected for temperature and viscosity effects and are reported for 25 °C. Hydrodynamic radii, R_h_, were estimated according to ${R}_{h}=\frac{kT}{6\pi {\rm{\eta }}{\rm{D}}}$ where k is Boltzmann’s constant, T is the system temperature and η is the solution viscosity. Power series were performed in order to determine the photobleaching limits. A triplet-state contribution of 1 μs with the expected irradiance-dependent amplitude was observed in all cases. All measurements were repeated at least 20 times and curves distorted due to occasional transits of big aggregates were excluded. The surfactant was added to the diluted vesicle samples immediately before the FCS measurement. Error bars indicate the standard error of the mean.

### Single-vesicle TIRF spectroscopy

Fluorescence emission at the donor and acceptor wavelengths were acquired from single vesicles by using a prism-type total internal reflection fluorescence microscope equipped with green (532 nm) and red (635 nm) lasers (Crystalaser, USA). Microscope slides were successively treated with biotinylated poly-ethyleneglycol (PEG) and NeutrAvidin, before pM concentrations of fluorescently-labelled vesicles were added. Fluorescence trajectories were acquired with an integration time of 50 ms. The base buffer used for imaging was 50 mM Tris (pH 8), 6% (w/v) glucose, 165 U/mL glucose oxidase, 2170 U/mL catalase and 2 mM trolox. Specified concentrations of TX-100 were included in imaging buffer prior to being injected into the sample. Spatially-separated fluorescence images of donor and acceptor emission were collected in custom built relay optics with a 550 nm long-pass filter and imaged in parallel using an EMCCD camera (iXON, Andor Technology). All measurements were performed at room temperature. SvFRET efficiency after background correction was approximated by the apparent FRET efficiency, E_FRET_ = (I_A_/[I_A_ + I_D_]) ~ R_o_^6^/([R_o_^6^ + R^6^]), where I_A_ and I_D_ are the fluorescence intensities of the acceptor and donor, respectively, R_o_ is the Forster radius and R is the separation distance between the probes. Since the quantum yields of Dil and DiD are similar, E_FRET_ closely matches the true efficiency of energy transfer. Half-lives were calculated by applying double exponential fits consisting of a rise (I = Ae^t/tE^) and decay (I = Be^−t/tL^) component to the donor trajectories. Data analysis was carried out using laboratory-written analysis routines developed in MATLAB 7. Ensemble information from svFRET measurements was obtained by assembling single-vesicle FRET trajectories into population FRET contour plots.

### Quartz crystal microbalance with dissipation (QCM-D) monitoring

Quartz crystal microbalance with dissipation monitoring (QCM-D) experiments were performed using a Q-sense E4 system (Biolin Scientific). SiO_2_-coated AT-cut quartz sensors (QSX 303, Biolin Scientific) were used, for which the fundamental frequency was 4.95 ± 0.05 MHz. The sensors were initially subjected to a 10 minute cleaning step by UV–ozone, prior to being sonicated in solutions of 2% Hellmanex III and 2x ultrapure Milli-Q water for 10 minutes. The sensors were then dried with N_2_ and placed under UV–ozone for a further 30 minutes. Each sensor was then immersed in 100% ethanol for 30 minutes and dried with N_2_ before installation in the flow modules. The QCM-D flow chambers were first flushed with ultrapure Milli-Q water for 1 hour, and then with 50 mM Tris buffer (pH 8) for 20–30 minutes before each measurement until a stable baseline was established (<0.5 Hz shift over 10 min). The flow rate was kept constant at 20 μL/min. The sensor surfaces were then functionalized with biotinylated polyethyleneglycol (Iris Biotech) which acts as a biocompatible support for specific immobilization of Avidin. The sensor surfaces were then rinsed with 50 mM Tris buffer (pH 8.0) for 15 min to remove unadsorbed molecules. Thereafter, Avidin was immobilized on the sensor surfaces by incubating a 0.1 mg/mL Avidin solution in 50 mM Tris buffer (pH 8.0) for 20 min, following a rinse step with 50 mM Tris buffer (pH 8.0) for 20 min to wash unbound Avidin molecules. Subsequently, vesicles coated with 1% biotinylated lipids were immobilized on the sensor surfaces by incubation with a 33 µg/mL vesicle solution for 70 min. Triton X-100 (TX-100) detergent solutions at specified concentrations were then introduced into the QCM-D flow chambers. Changes in mass (∆m) were related to changes in frequency (∆f) via the Sauerbrey equation ∆m = −(C · ∆f)/n where n is the overtone number and C is a constant related to the properties of the quartz (17.7 ng Hz^−1^ cm^−2^).

## Supplementary information

Supplementary Information

## Data Availability

The datasets generated during the current study are available from the corresponding author on reasonable request.
