# Supplementary material for: Unveiling the multi-step solubilization mechanism of sub-micron size vesicles by detergents
Source: Sci Rep. 2019 Sep 9;9:12897. doi: 10.1038/s41598-019-49210-0 (PMC6733941; doi:10.1038/s41598-019-49210-0)
Supplement: Supplementary file 1 — Supplementary Information [file 41598_2019_49210_MOESM1_ESM.pdf]

Unveiling the multi-step solubilization mechanism of sub-micron size vesicles by detergents

Paul A. Dalgarno[a,b], José Juan-Colás[c], Gordon J. Hedley[a¶], Lucas Piñeiro[d], Mercedes Novo[d], Cibran Perez-Gonzalez[a], Ifor D. W. Samuel[a], Mark C. Leake[e,f], Steven Johnson[c], Wajih Al-Soufi[d], J. Carlos Penedo[*a,g] & Steven D. Quinn[*a,e,f]

[a] SUPA School of Physics and Astronomy, University of St. Andrews, North Haugh, Fife, UK. KY16 9SS.

[b] Institute of Biological Physics and Bioengineering, School of Engineering and Physical Sciences, Heriot-Watt University, Edinburgh, UK. EH14 4AS.

[c] Department of Electronic Engineering, University of York, Heslington, York, UK. YO10 5DD.

[d] Department of Physical Chemistry, Faculty of Science, University of Santiago de Compostela, Lugo, Spain. E-27002.

[e] Department of Physics, University of York, Heslington, York, England, UK. YO10 5DD.

[f] Department of Biology, University of York, Heslington, York, UK. YO10 5DD.

[g] Biomedical Sciences Research Complex, University of St. Andrews, North Haugh, St. Andrews, Fife, UK. KY16 9ST.

*Corresponding authors: (SDQ) steven.quinn@york.ac.uk and JCP (jcp10@st-andrews.ac.uk).

¶ Present Address: School of Chemistry, University of Glasgow, Glasgow, Scotland, United Kingdom. G12 8QQ.

SUPPORTING INFORMATION

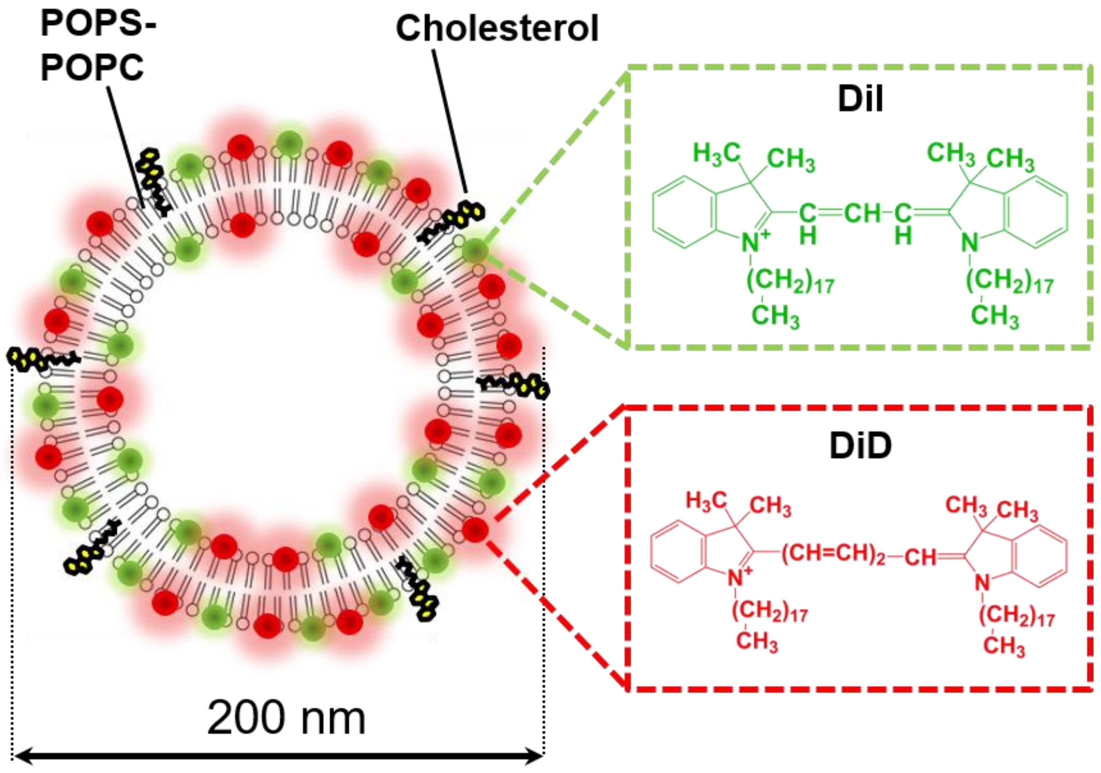

Figure S1. Schematic representation of PS/PC vesicles fluorescently-labelled with DiI and DiD. Insets: chemical structures of DiI (green) and DiD (red). DiI, acting as the FRET donor, and DiD, acting as the FRET acceptor are derivatives of the widely used Cy3 and Cy5 dyes (Ro ~ 53 Å) in which the chain between aromatic groups has been extended to enable their incorporation within a lipid bilayer.

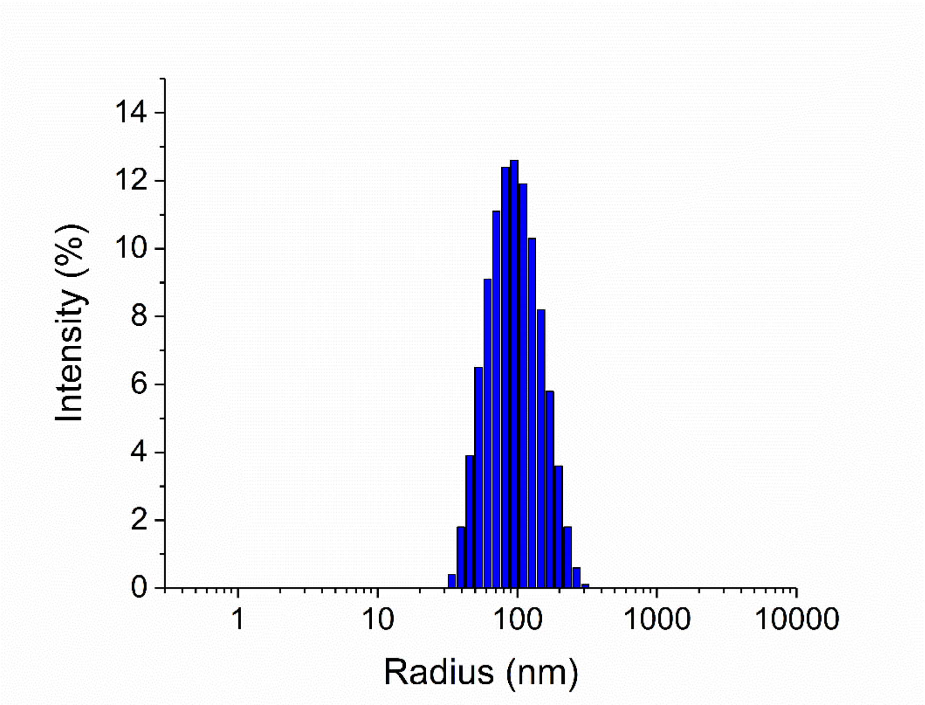

Figure S2. Semi-log plot of vesicle radius as a function of scattering intensity, as obtained from dynamic light scattering. Experimental conditions: Tris-HCl buffer, pH 7.9, 21ºC.

Table S1 Pre-exponential factors, fluorescence lifetimes and fitting errors associated with DiI in the presence of the acceptor DiD obtained from freshly prepared PC/PS vesicles containing 20 % cholesterol as a function of TX-100. Experimental conditions: Tris-HCl buffer, pH 7.9, 21ºC.

	**0 mM**	**0.15 mM**	**0.30 mM**	**0.45 mM**	**1.00 mM**	**4.00 mM**
**a_1**	0.35 ± 0.02	0.29 ± 0.01	0.26 ± 0.01	0.27 ± 0.02	0.18 ± 0.01	0.12 ± 0.01
**τ_1 / ps**	18 ± 1	140 ± 7	150 ± 7.5	200 ± 10	150 ± 7.5	121 ± 6
**a_2**	0.80 ± 0.02	0.71 ± 0.04	0.74 ± 0.04	0.73 ± 0.04	0.82 ± 0.04	0.88 ± 0.04
**τ_2 / ps**	615 ± 7	890 ± 44.5	940 ± 47	1000 ± 50	1150 ± 58	1250 ± 63
**τ_{av}^*/ ps**	***453 ± 5***	***672 ± 34***	***735 ± 37***	***784 ± 39***	***970 ± 49***	***1110 ± 56***

* Amplitude weighted average lifetimes, τ_{av}, were calculated according to the equation $\tau_{av} = \sum_{i=1}^{N} a_i \tau_i^2 / \sum_{i=1}^{N} a_i \tau_i$ where a_i represents the pre-exponential factor and τ_i the associated fluorescence lifetime.

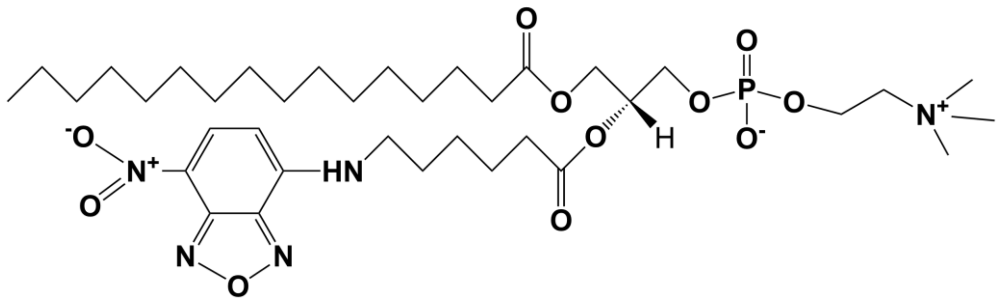

Figure S3. Chemical structure of NBD-PC.

Table S2 Summary of the diffusion times determined by the correlation curves for each FCS measurement. The calculated values for the size of the focus, diffusion time, the diffusional coefficient and diameter for each species are shown.

Condition	Focus (μm)	t_D (μs)	D ($x10^{-10} m^2 s^{-1}$)	d (nm)
**Rh123**	0.27	40 ± 1	4.60 ± 0.4	1.06 ± 0.09
**100 nm LUVs**	0.27	6100 ± 30	0.030 ± 0.003	163 ± 16
**100 nm LUVs + 0.1 mM TX-100**	0.27	6900 ± 300	0.027 ± 0.003	184 ± 17
**100 nm LUVs + 0.2 mM TX-100**	0.27	8200 ± 500	0.022 ± 0.003	219 ± 25
**200 nm LUVs**	0.27	8000 ± 300	0.023 ± 0.002	212 ± 20
**200 nm LUVs + 0.1 mM TX-100**	0.27	8600 ± 100	0.021 ± 0.002	230 ± 20
**200 nm LUVs + 0.2 mM TX-100**	0.27	9400 ± 600	0.019 ± 0.002	253 ± 27

Supplementary Text I

Optimization of Vesicle Photostability for svFRET Experiments

To optimize conditions necessary for FRET based vesicle-detergent experiments, the photobleaching rates of DiD-labelled PC/PS vesicles were investigated. The goal was to determine the factors that modulate acceptor photostability, and, to identify methods to minimise their effects. A schematic illustration of the vesicles is shown in **Figure S4 a**. The vesicles were studied using TIRF spectroscopy with λ_{ex} = 635 nm as a function of excitation intensity (0.04 mW/cm^2, 0.4 mW/cm^2 and 0.8 mW/cm^2) and molar percentage of labelling (0.05 % - 0.5 %). In the absence of TX-100, DiD-labelled vesicles displayed photobleaching rates that were faster than those associated with single dyes, which we attributed to dye crowding on the membrane surface. For example, Cy5 fluorophores covalently attached to immobilized single-stranded DNA molecules remained photoactive for a period of ~60 seconds under an excitation intensity of 0.4 mW/cm^2 before displaying single-step photobleaching (**Figure S4 b**). In contrast, the fluorescence intensity from DiD-labelled vesicles was found to display increasing levels of destabilization as the excitation power increased from 0.04 to 0.8 mW/cm^2, though the effect was lessened by reducing the dye-loading on each vesicle from 0.5 % to 0.05 %. When the labelling density was 0.5 % and the excitation intensity set to 0.4 mW/cm^2, the DiD fluorescence trajectories displayed bi-exponential behaviour with an average decay time of 28 ± 4 s. As the excitation intensity decreased to 0.04 mW/cm^2, the fluorescence decays were dominated by longer lived components and an average decay time of 152 ± 7 s was observed. With a labelling concentration of 0.05 %, the average decay time at 0.4 mW/cm^2 was 35 ± 3 s and increased to 439 ± 10 s when the excitation power decreased ten-fold. At 0.8 mW/cm^2, the fluorescence trajectories decayed bi-exponentially with average decay times of 13 ± 1 s and 14 ± 1 s when labelling concentrations of 0.5 and 0.05 % were tested. Representative photobleaching traces of

DiD labelled vesicles as a function of excitation intensity and dye loading are shown in **Figure S4 c-e**. Pre-exponential factors and kinetics of the photobleaching trajectories under the same conditions are shown in **Table S3**.

When imaging of labelled vesicles containing 0.125 % DiI and 0.125 % DiD, as necessary for svFRET experiments, was performed, an excitation intensity of < 0.04 mW/cm^2 was thus required for long-term photostability. Under this condition, the fluorescence trajectories of both dyes, and therefore the corresponding efficiency of energy transfer, were found to be invariant over the measurement time window as demonstrated by the representative fluorescence trajectories shown in **Figure S4 f, g**. An analysis of N > 500 vesicles revealed good photostability over a 3 minute time window, as demonstrated by a lack of variation in the FRET contour plot shown in **Figure S4 h.** Given that the effect of photobleaching was minimised, this provided an opportunity to study vesicle perturbation by TX-100 using svFRET as the optical sensor.

Cy5-ssDNA Labelling Reaction, Precipitation and Purification

C20-3'amino DNA was labelled with the DiD analogue Cy5 (Integrated DNA Technologies, IA, USA) for single-molecule analysis. Here, the dry pellet of DNA was dissolved in deionised water to a final concentration of 25 μg/μL. Cy5 was suspended in dimethyl sulfoxide (DMSO) to a final concentration of 22.5 mM, dried using a SpeedVac system for 4 hours and re-suspended in a solution composed of 75% (v/v) labelling buffer (0.1 M sodium tetraborate, pH 8.5, 7% (v/v) deionised water, 14% (v/v) DMSO and 4 % (v/v) C20-3'amino DNA). This was stored in darkness at room temperature under gentle agitation for 16 hours. Sodium acetate (0.3 M) and ice cold absolute ethanol (250 μL) were subsequently added to the labelling reaction, mixed gently by inversion and incubated at -20°C for a further 16 hours. After incubation, the solution

was centrifuged at 13000 rpm for 1 hour and the supernatant removed. The remaining pellet was rinsed with cold ethanol, dried with nitrogen and dissolved in 50 mM Tris-HCl buffer (pH 7.5). Denaturing polyacrylamide gel electrophoresis was subsequently performed at 4°C to separate labelled and unlabeled DNA. Final Cy5-DNA constructs were stored at 4°C prior to use.

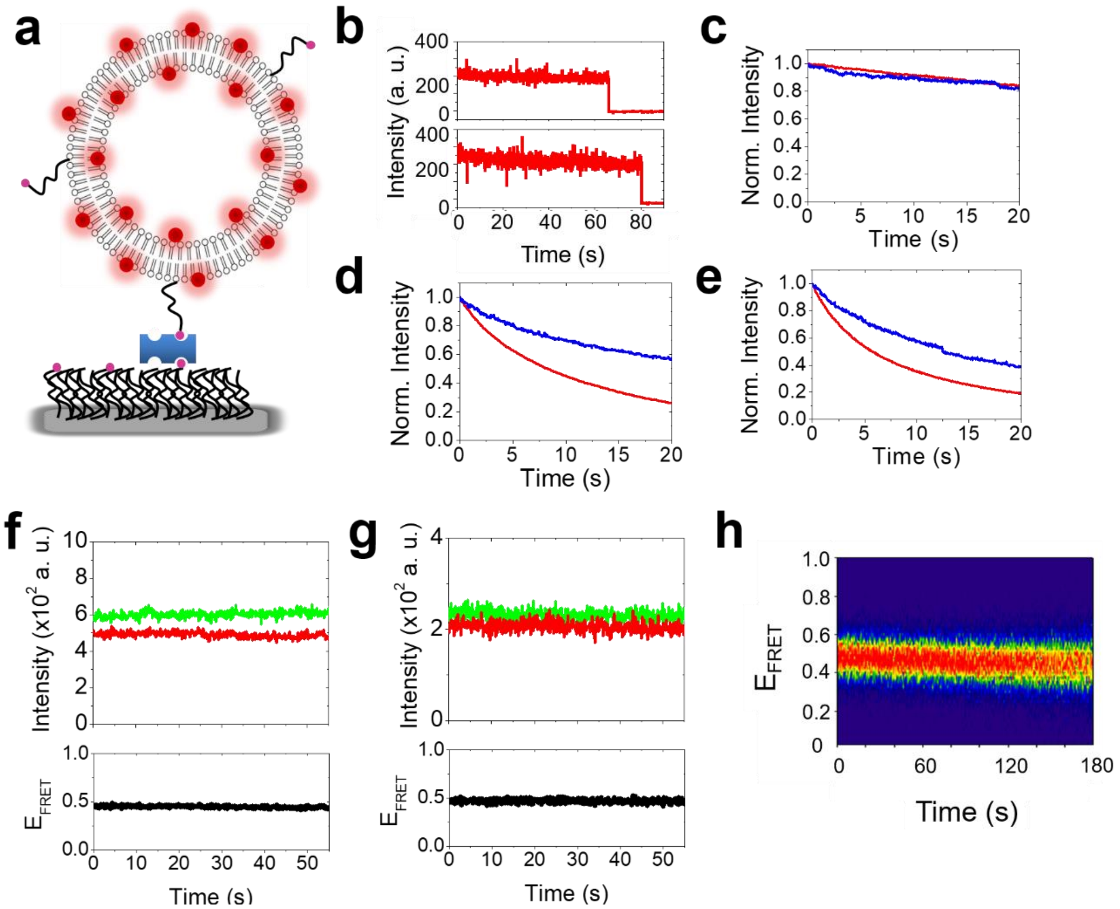

Figure S4. Optimization of vesicle photostability. (a) Schematic of the immobilization scheme. (b) Representative stepwise photobleaching trajectories obtained from Cy5-labelled ssDNA under continuous excitation of 0.4 mW/cm^2 with λ_{exc} = 635 nm. The normalised sum of intensity trajectories from N >1000 individual vesicles labelled with 0.5 % (red) and 0.05 % (blue) DiD are shown for excitation intensities of (c) 0.04 mW/cm^2, (d) 0.4 mW/cm^2 and (e) 0.8 mW/cm^2 (λ_{exc} = 635 nm). (f, g) Representative fluorescence trajectories from LUVs composed of 0.125 % DiI (green) and 0.125 % DiD (red) with an excitation intensity < 0.04 mW/cm^2. Also shown are the corresponding FRET trajectories (λ_{exc} = 532 nm). (h) FRET contour plot extracted from N > 500 vesicles as described with an excitation intensity < 0.04 mW/cm^2.

Table S3. Pre-exponential factors, lifetimes and fitting errors associated with vesicles composed of 0.5 % and 0.05 % DiD as a function of excitation power. Experimental conditions: Tris-HCl buffer, pH 7.9, 21°C.

	**0.04 W/cm^2**		**0.4 W/cm^2**		**0.8 W/cm^2**	
[DiD] (molar percent)	**0.5 %**	**0.05 %**	**0.5 %**	**0.05 %**	**0.5 %**	**0.05 %**
**a_1**	0.13 ± 0.01	0.20 ± 0.01	0.09 ± 0.01	0.13 ± 0.01	0.34 ± 0.01	0.38 ± 0.02
τ_1 / s	24.2 ± 0.3	18.7 ± 0.3	1.8 ± 0.1	3.6 ± 0.1	3.1 ± 0.1	14.2 ± 0.7
**a_2**	0.69 ± 0.01	1.04 ± 0.01	0.50 ± 0.01	0.68 ± 0.01	0.61 ± 0.01	--
τ_2 / s	155.6 ± 7.3	447 ± 9.8	8.4 ± 0.1	35.7 ± 3.3	13.8 ± 0.1	--
**a_3**	--	--	0.43 ± 0.01	--	--	--
τ_3 / s	--	--	34.4 ± 5.7	--	--	--
**τ_{av}*/ s**	***151.9 ± 7.1***	***438.6 ± 9.7***	***28.4 ± 4.4***	***35.1 ± 3.2***	***12.6 ± 0.1***	***14.2 ± 0.7***

* Amplitude-weighted average lifetimes were calculated according to the equation $\tau_{av} = \sum_{i=1}^{N} a_i \tau_i^2 / \sum_{i=1}^{N} a_i \tau_i$ where a_i represents the pre-exponential factor and τ_i the associated decay lifetime.

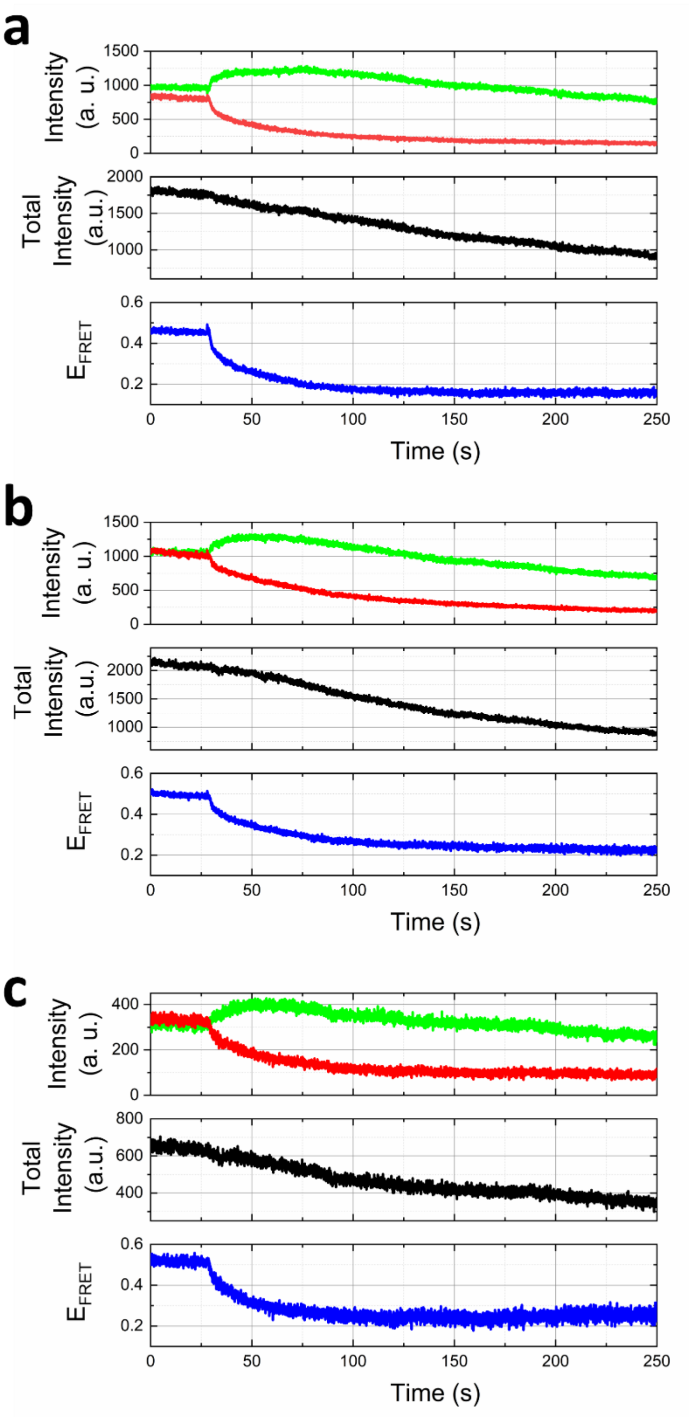

Figure S5. Real-time visualization of TX-100 induced vesicle solubilization kinetics by svFRET. (a-c) Representative variation in the fluorescence emission of DiI (green) and DiD (red) (top panel), the sum of their intensities (middle panel) and the corresponding variation in FRET efficiency obtained before (< 25 s) and after (> 25 s) injection of 0.16 mM TX-100 into immobilized vesicles containing 20 % cholesterol.

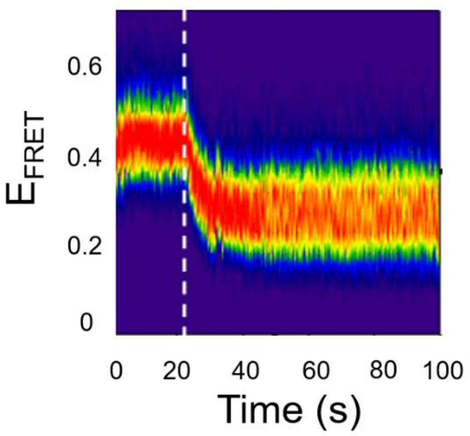

Figure S6 TX-100 induced vesicle expansion probed by svFRET. FRET contour plot showing the variation in FRET efficiency as a function of time when 0.16 mM TX-100 is injected (dashed white line) into immobilized DiI/DiD labelled PS/PC vesicles in the absence of cholesterol.

Supplementary Text II

Vesicle solubilization monitored by Quartz Crystal Microbalance with Dissipation Monitoring (QCM-D)

Quartz crystal microbalance with dissipation monitoring (QCM-D) was employed to investigate conformational changes of vesicles immobilized to a gold coated quartz sensor substrate. Here, vesicles containing a base ratio of 65: 35 PC: PS incorporating 20 % cholesterol and 1 % biotinylated lipids were immobilized onto the QCM-D sensor surface as described in the Methods section. Interactions between the vesicles and TX-100 were monitored through real-time changes in oscillation frequency and dissipation, reflecting the mass and rigidity of the immobilized vesicles, respectively, as TX-100 was flushed over the sensor surface. Upon exposure of the vesicles to TX-100 concentrations < 0.16 mM solution, a small decrease in frequency was consistently observed in all harmonics which we attribute

to the interaction of the detergent molecules with the lipid layer of the vesicles. At higher TX-100 concentrations, a pronounced interaction of the detergent with the surface-immobilized vesicles was observed as demonstrated by both frequency and dissipation responses at the sensor. The ripple observed in both frequency and dissipation traces immediately after TX-100 was introduced is attributed to two events: first, the deposition of TX-100 molecules on the vesicle membrane, and second, a non-disruptive interaction of the detergent molecules with the vesicles that leads to re-structuring of the vesicle conformation (**Figure 3a**). Control experiments performed simultaneously indicate no interaction with the PEG-coated sensor surface and TX-100 at the concentrations tested, as indicated by the lack of variation in the frequency and dissipation response after TX-100 injection. As TX-100 builds up, the deposited mass continues to increase leading to a decrease in resonance frequency. Subsequently, following a steep transition which indicates re-structuring of the vesicles, an increase in resonance frequency (i.e. decrease in mass) is observed in the harmonics of the resonant wave, indicating material immobilized to the surface, namely lipid and aqueous solution encapsulated by the vesicles, is released into the solution following TX-100 accumulation. The energy dissipation response supports these deductions, as prior to the steep transition there is an increase in energy dissipation, which relates to the rigidity decrease obtained from the deposition of TX-100 molecules. After a sharp, short transition, the dissipation levels are decreased indicating an increase in rigidity due to the incorporation of TX-100 onto the vesicles. This change in vesicle conformation also leads to a cross in the different harmonic dissipation trends of the sensor as they have a different penetration depth and therefore changes in conformation affect the energy dissipation response in different manners. The interaction of the TX-100 detergent at this concentration with the surface-immobilized vesicles can be observed more clearly by plotting the energy dissipation response against the change in resonance frequency for a given overtone (here the 7th

overtone) as shown in **Figure 3b**. An interaction between TX-100 and immobilized vesicles can be deduced from the QCM-D sensing strategy whereby TX-100 binds to the surface-immobilized vesicles initiating a structural reorganization consistent with vesicle expansion, which precedes a major lysis event. When complete solubilization was achieved, the frequency and dissipation responses closely matched those obtained from a control sensor lacking vesicles.
